# Development of a Gastrointestinal-Myoelectrical-Activity-Based Nomogram Model for Predicting the Risk of Mild Cognitive Impairment

**DOI:** 10.3390/biom12121861

**Published:** 2022-12-12

**Authors:** Baichuan Li, Shuming Ji, Anjiao Peng, Na Yang, Xia Zhao, Peimin Feng, Yunwu Zhang, Lei Chen

**Affiliations:** 1 Department of Neurology, Joint Research Institution of Altitude Health, West China Hospital, Sichuan University, Chengdu 610044, China; 2 Department of Clinical Research Management, West China Hospital, Sichuan University, Chengdu 610044, China; 3 Department of Gastroenterology, Hospital of Chengdu University of Traditional Chinese Medicine, Chengdu 610044, China; 4 Fujian Provincial Key Laboratory of Neurodegenerative Disease and Aging Research, Institute of Neuroscience, School of Medicine, Xiamen University, Xiamen 361005, China

**Keywords:** mild cognitive impairment, Alzheimer’s disease, electrogastroenterography, LASSO regression, nomogram, decision curve analysis

## Abstract

Mild cognitive impairment (MCI) is the prodromal stage and an important risk factor of Alzheimer’s disease (AD). Interventions at the MCI stage are significant in reducing the occurrence of AD. However, there are still many obstacles to the screening of MCI, resulting in a large number of patients going undetected. Given the strong correlation between gastrointestinal function and neuropsychiatric disorders, the aim of this study is to develop a risk prediction model for MCI based on gastrointestinal myoelectrical activity. The Mini-Mental State Examination and electrogastroenterography were applied to 886 participants in western China. All participants were randomly assigned to the training and validation sets in a ratio of 7:3. In the training set, risk variables were screened using LASSO regression and logistic regression, and risk prediction models were built based on nomogram and decision curve analysis, then validation was performed. Eight predictors were selected in the training set, including four electrogastroenterography parameters (rhythm disturbance, dominant frequency and dominant power ratio of gastric channel after meal, and time difference of intestinal channel after meal). The area under the ROC curve for the prediction model was 0.74 in the training set and 0.75 in the validation set, both of which exhibited great prediction ability. Furthermore, decision curve analysis displayed that the net benefit was more desirable when the risk thresholds ranged from 15% to 35%, indicating that the nomogram was clinically usable. The model based on gastrointestinal myoelectrical activity has great significance in predicting the risk of MCI and is expected to be an alternative to scales assessment.

## 1. Introduction

Alzheimer’s disease (AD) is the most common type of dementia and has become one of the most lethal and burdensome diseases this century [1]. Mild cognitive impairment (MCI) is the prodromal stage and most important independent risk factor of AD [2,3]. More than 50% of MCI patients develop AD within 5 years [4], with a conversion rate 10–15 times higher than that of normal older adults [5]. Studies have shown that, unlike in AD, where they have limited efficacy, cognitive training and exercise therapy for MCI patients can obtain significant results [6,7]. Therefore, enhancing the early screening, diagnosis, and treatment of MCI is particularly important to reduce the occurrence of AD and the burden of disease.

Diagnosis of MCI is a step-by-step process, which is usually first identified in the community or primary care center and subsequently confirmed in a neurological specialty center [8]. The most common method used in screening is neuropsychological testing, such as the Mini-Mental State Examination (MMSE) scale [9]. However, scale assessment is time-consuming and with subjective results, and many participants are also unable to complete the questionnaire effectively due to cultural differences and literacy levels [10,11,12], resulting in a large number of MCI patients in the community remaining undetected [13]. Thus, objective, convenient, and efficient screening methods for MCI are urgently needed.

Traditional medicine in China has long had the theory of “disorder of the stomach leading to insomnia with restlessness” and “if the stomach is dry and the stool is hard, one is delirious”. Similarly, Western medicine also has the concept of the gut–brain axis, all of which suggest an important connection between gastrointestinal homeostasis and the central nervous system (CNS) [14]. Numerous studies have shown that gastrointestinal dysfunction affects the development of CNS disorders (e.g., autism, Parkinson’s disease, schizophrenia) [15,16,17,18]. Recent studies have also found that inflammatory bowel disease (IBD) increases the risk of dementia and that dementia occurs earlier and more severely as gastrointestinal symptoms deteriorate [19], suggesting a correlation between gastrointestinal function and dementia or cognitive dysfunction.

Electrogastroenterography (EGEG) is a non-invasive method of recording gastrointestinal myoelectrical activity using electrodes placed on the skin of the abdomen. It was first discovered in 1921 and was widely used in clinical practice in the 1990s [20]. Because of the advantage of its objective, stable, and non-invasive reflection of gastrointestinal slow waves, EGEG has become an attractive and powerful tool for examining the gastrointestinal function of digestive diseases or other diseases that are prone to gastrointestinal discomfort (e.g., depression and anxiety [21], Parkinson’s [22], obesity [23]). However, it is unclear whether these parameters can be used to reflect neurological function or neuropsychiatric disorders.

Therefore, the aim of this study is to develop a risk prediction model for MCI based on gastrointestinal myoelectrical activity. The novel eight-channel EGEG was used to record gastrointestinal myoelectrical activity in 886 subjects from 60 communities in western China, and we found that EGEG can help screen for MCI and is expected to be a complement to cognitive assessment scales.

## 2. Materials and Methods

### 2.1. Participants

Participants older than 40 from 60 communities in western China were recruited from October 2019 to October 2021. General information on age, sex, marital status, education level, lifestyle, dietary habits, and family history was collected; routine blood tests and biochemical tests were conducted; and MMSE assessment and EGEG detection were conducted. Participants who were deaf or blind, diagnosed with gastrointestinal diseases such as gastritis or gastric ulcer within the previous six months, or with gastrointestinal discomforts such as diarrhea or constipation were excluded, and those who had severe organ insufficiencies such as of the heart, liver, or kidney or metabolic diseases such as diabetes were also excluded. In addition, participants whose parents or siblings had early onset dementia or mild cognitive impairment (e.g., at less than 30 years old) were excluded. To reduce the effect of drugs on gastrointestinal electrogram testing, those who had taken any drugs one week before the examination were excluded. This study was approved by the Ethics Committee of West China Hospital of Sichuan University (Nos. 2018-491 and 2022-1137).

### 2.2. Cognitive Function Assessment

The MMSE was used to assess the cognitive function of the participants [24]. The scales have good reliability and validity and were conducted by two professionals in a quiet, comfortable setting and with uniform terminology guidance.

### 2.3. EGEG Records

**Figure 1:**
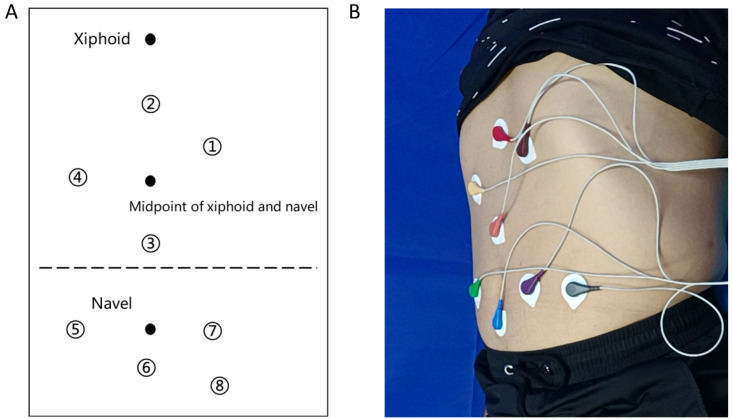
(**A**) A pattern diagram of electrode positioning for EGEG recording. The eight electrodes represent eight channels used to reflect the myoelectrical activity of the corpus gastricum, lesser curvature, greater curvature, antrum, ascending colon, transverse colon, descending colon, and rectum. (**B**) A diagram of the process in EGEG examination. EGEG, electrogastroenterogram.

Gastrointestinal myoelectrical activity was measured using an eight-channel gastrointestinal electromyograph as described previously (XDJ-S8, Hefei Kaili Co., Hefei, China) [25]. Participants were told to avoid alcohol and spicy or irritating foods for at least three days and to fast for more than 6 h before the examination. Measurements were performed in the supine position (Figure 1). Four gastric electrodes (reflect gastric body, antrum, lesser gastric curvature, greater gastric curvature) and four intestinal electrodes (reflect ascending colon, transverse colon, descending colon, rectum) were placed on the abdominal skin (Hanjie Co., Ltd., Shanghai, China). The earth electrode was placed on the medial ankle of the right calf and the reference electrode was placed on the medial wrist of the right hand. The participants were informed to be quiet and not move or talk during the examination. Besides this, a mealtime functional load test was performed with about 200 kcal of food after a 6 min preprandial EGEG recording.

### 2.4. Gastrointestinal Electrical Index

In order to filter out the background noise, the EGEG sampling rate was 1 HZ and the filtering frequency was between 0.008 HZ and 0.1 HZ. The following parameters were derived through software for spectral analysis: (1) mean amplitude (MA); (2) average frequency (AF); (3) rhythm disturbance (RD); (4) reactive area (RA); (5) time difference of the channel (TD); (6) dominant frequency (DF); (7) dominant power ratio (DP); (8) percentage of normal slow wave (PNSW); and (9) coupling percent (CP).

Given that we recorded four gastric channels and four intestinal channels, and that high correlations among the four channels in the gastric region and among the four channels in the intestinal region were found in a previous study (data not shown), we merged the four gastric channels into one gastric channel and the four intestinal channels into one intestinal channel in this study. Because we also recorded both before- and after-meal data, a total of 36 parameters were derived for each participant.

### 2.5. Statistical Analysis

The statistical analysis in our study consisted of two parts: variable selection and an assessment of predictive power. In order to obtain the subset of predictors, we used least absolute shrinkage and selection operator (LASSO) regression analysis to select variables. Furthermore, the LASSO regression analysis was run with 10-fold cross-validation for centralization and normalization of the included variables, and then “lambda.min” was used to select those with the best performance. Participants in our study were randomly divided into a training set and a validation set in a theoretical ratio of 7:3. Stepwise multi-variable logistic regression analysis was used to build a predicting model by introducing the predictors selected in the LASSO regression model in the training set. The statistically significant predictors were retained finally and were applied to produce a prediction model for MCI risk by developing a nomogram prediction model.

Further, several kinds of validation methods were used to estimate the accuracy of the risk prediction model by using the data of the training set and the validation set. The area under the receiver operating characteristic (ROC) curve was used to provide good discrimination for the quality of the risk nomogram to separate true positives from false positives. The calibration curve was used to evaluate the calibration of the MCI risk nomogram, accompanied by the Hosmer–Lemeshow test. Decision curve analysis was used to determine the clinical practicability of nomograms based on the net benefit under different threshold probabilities in the natural population cohort. All analyses were performed using R version 4.1.3 with packages glmnet and rms, and the significance level was set as a two-tailed alpha of <0.1.

## 3. Results

### 3.1. Clinical Baseline Information of Participants

A total of 886 participants were recruited, including 273 males and 613 females, and 106 participants were diagnosed with MCI (106/886, 12.0%). The participants were randomly assigned in a ratio of 7:3, and 620 and 266 participants were thus assigned to the training and validation sets, respectively. The relevant clinical baseline information for both groups is shown in Table 1, and comparisons of the 36 EGEG parameters are exhibited in Supplementary Table S1. The results showed that age, high-density lipoprotein (HDL), and dominant frequency of gastric channel after meal (DFGA) were significantly different between MCI patients and non-MCI subjects in both sets (*p* < 0.001). 

### 3.2. Independent Risk Factor Screening

**Figure 2:**
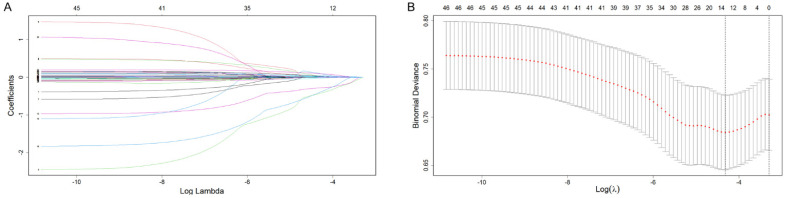
The process of variable selection by the LASSO binary logistic regression model. A coefficient profile plot was produced against the log(lambda) sequence (**A**). Thirteen variables with non-zero coefficients were selected by optimal lambda. By verifying the optimal lambda in the LASSO model, the partial likelihood deviance (binomial deviance) curve was plotted. Different colors represent the different variables. (**B**), and dotted vertical lines were drawn based on minimum lambda and standard error criteria.

Among the 46 associated variables, 13 potential predictor variables were finally retained in the training set based on the non-zero coefficient characteristic variable screening process of LASSO regression (Figure 2). These predictor variables were age, alcohol, body mass index (BMI), HDL, low-density lipoprotein (LDL), percentage of normal slow wave of gastric channel before meal (PGB), dominant power ratio of gastric channel before meal (DPGB), dominant frequency of gastric channel after meal (DFGA), mean frequency of gastric channel after meal (MFGA), rhythm disturbance of gastric channel after meal (RDGA), dominant power ratio of gastric channel after meal (DPGA), rhythm disturbance of intestinal channel after meal (RDIA), and time difference of intestinal channel after meal (TDIA).

### 3.3. Building the Predictive Model

**Figure 3:**
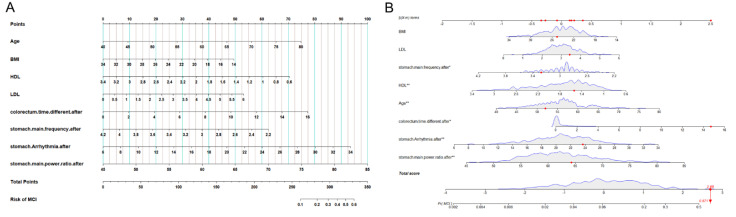
Development of the MCI risk nomogram in the training set (**A**) and in the validation set (**B**). The MCI risk nomogram was developed with the predictors age, BMI, HDL, LDL, TDIA, DFGA, RDGA, and DPGA. BMI, body mass index; HDL, high−density lipoprotein; LDL, low−density lipoprotein; TDIA, time difference of intestinal channel after meal; DFGA, dominant frequency of gastric channel after meal; RDGA, rhythm disturbance of gastric channel after meal; DPGA, dominant power ratio of gastric channel after meal. *: *p* < 0.05, **: *p* < 0.01.

To select the optimal characteristic variables, stepwise logistic regression was used for further analysis. Eight predictor variables were selected through the regression: age, BMI, HDL, LDL, DFGA, RDGA, DPGA, and TDIA, as shown in Table 2. All of the variables were significantly different at the 0.1 test level. Based on the results above, the risk prediction model for cognitive dysfunction was built and the nomograms were plotted (Figure 3); these are helpful for clinical evaluation and individualized prediction.

### 3.4. Validation of the Predictive Model

**Figure 4:**
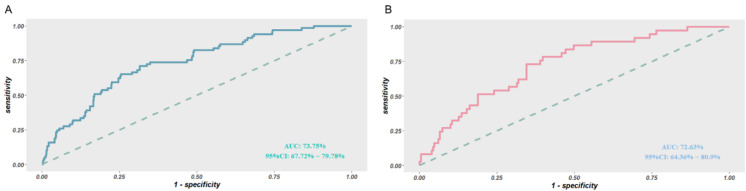
ROC curves of the MCI risk nomogram prediction in the training set (**A**) and in the validation set (**B**). The y-axis indicates the true-positive rate of the risk prediction. The x-axis indicates the false-positive rate of the risk prediction.

**Figure 5:**
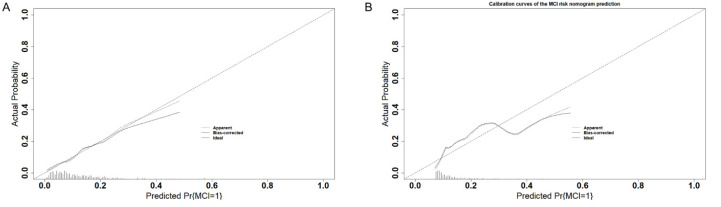
Calibration curves of the MCI risk nomogram prediction. The y-axis indicates the actual diagnosed MCI. The x-axis indicates the predicted risk of MCI. The diagonal dotted line indicates perfect prediction by an ideal model. The solid line represents the performance of the training set (**A**) and validation set (**B**); a closer fit to the diagonal dotted line represents a better prediction.

**Figure 6:**
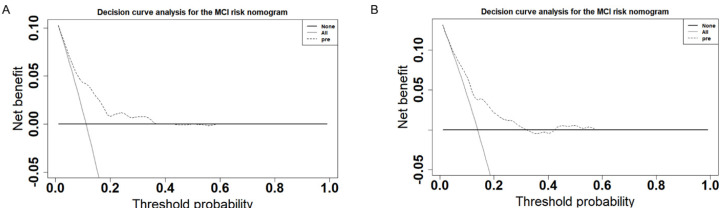
Decision curve analysis for the MCI risk nomogram in the training set (**A**) and in the validation set (**B**). The y−axis measures the net benefit. The thick solid line represents the assumption that no patients have MCI. The thin solid line represents the assumption that all patients have MCI. The dotted line represents the risk nomogram.

First, we explored the efficiency of the nomograms. The results showed that the area under the ROC curve of the nomograms was 0.74 (95% CI: 0.68–0.80) in the training set and 0.81 (95% CI: 0.74–0.88) in the validation set (Figure 4), both of which exhibited great performance. This also shows good consistency in the calibration curves of the nomograms for both sets (Figure 5). However, as the predicted risk probability rose above 0.4, the accuracy of the model prediction started to perform more significantly lower than the model fit results on the validation set, suggesting that the predictive ability of the model may be compromised in the high-risk segment. Furthermore, decision curve analysis was conducted (Figure 6), and the results revealed that in the training set, the risk of MCI could be predicted more accurately using the nomograms when the risk threshold probability of the model ranged between 12% and 35%. In the validation set, the threshold range should be controlled between 15% and 40%. Taken together, the net benefit of controlling the threshold range between 15% and 35% was more desirable and comparable. 

## 4. Discussion

MCI is an important period for dementia prevention and treatment [26]; improving its diagnosis rate can help delay the development of dementia and reduce the impact of dementia on people’s quality of life, along with its economic burden. However, there is no consensus on guidelines for routine screening for MCI, resulting in a large number of MCI patients in the community going undiagnosed. Therefore, it is significant to develop a convenient, efficient, and stable screening tool for MCI. In this study, we recruited 886 subjects from 60 communities in western China; their cognitive function was assessed via the MMSE scale, and gastrointestinal myoelectrical activity was measured via EGEG. Furthermore, a statistical model for MCI risk prediction was established based on gastrointestinal electrical parameters, and we found that the model had a good and stable predictive effect on MCI. 

In a recent epidemiological survey [27], MCI was diagnosed using the expanded Mayo Clinic criteria; the results showed that the mean prevalence of MCI was approximately 18.9% among different countries, with the lowest rate in Italy (7.7%) [28] and the highest rate in France (42.0%) [29]. Of the 886 participants from western China included in this study, 106 were found to have MCI, with an incidence of 12%, which is consistent with the previous findings. We assessed MCI using the MMSE scale, which is one of the most common cognitive screening tools used by general practitioners and specialists in Western countries. The scale can simply assess multiple cognitive domains with a sensitivity of 71% and a specificity of 74% [30]. Although the scale is widely used, it still has the disadvantages of being subjective and time-consuming, and many participants are also unable to complete the questionnaire effectively due to cultural differences and literacy levels [31]. In addition, there are also studies suggesting the use of scales such as the Informant Questionnaire on Cognitive Decline in the Elderly (IQCODE) and Montreal Cognitive Assessment (MoCA) for MCI screening, which also face problems such as strong subjectivity.

EGEG, similar to electrocardiograph (ECG) and electroencephalogram (EEG), is a method of recording gastrointestinal muscle electrical activity on the body surface of the human abdominal wall using skin electrodes, which is convenient, non-invasive, and objective [32]. Because the gastrointestinal electrical signal is very weak and slow, and its amplitude is only 1/1000 that of ECG, the research on gastrointestinal electrograms started late and has developed slowly [33]. EGEG is now used as a routine clinical aid in diagnosis. A large number of studies have shown that EGEG has good diagnostic value for gastrointestinal diseases such as gastritis, gastric ulcer, and tachycardia/bradycardia, and it also has some diagnostic significance for functional dyspepsia with obesity [23], motion sickness [34], and post-surgical gastrointestinal dysfunction [35].

Regarding the use of EGEG in neuropsychiatric disorders, most studies have taken EGEG as an adjunctive tool, for example, to assess whether an improvement in gastrointestinal discomfort contributes to an improvement in depressive and anxiety symptoms. Few studies have directly investigated whether EGEG can be used as an objective diagnostic tool. In this regard, the present study provides strong evidence that MCI patients without gastrointestinal discomfort showed significant signal changes on the EGEG, as evidenced by DFGA, RDGA, DPGA, and TDIA. These indicators are closely related to gastrointestinal function status: DFGA and DPGA can indirectly reflect the gastric electrical power, RDGA reflects the regularity of gastrointestinal electricity, and TDIA represents the electrical signal expansion rate. It can be seen that these four indicators can reflect gastrointestinal function to some extent. The high accuracy of this model in predicting MCI further suggests that gastrointestinal function status is highly correlated with MCI, and that gastrointestinal status can be used to reflect and judge MCI. 

Although the findings were derived from a multicenter study with a certain size, there are still some limitations. The biggest one is that the participants are mainly from western China, and whether the prediction model is applicable to MCI patients from other regions, other cultures, or other ethnicities remains unknown; this is expected to be confirmed in future work.

## 5. Conclusions

This study established a risk prediction model for MCI by combining LASSO regression and multiple stepwise regression, screened eight factors with a higher risk in the training set, and validated them in the test set to obtain better prediction results. This study is expected to provide an objective reference for MCI diagnosis, which is helpful to improving the diagnosis rate of MCI, delaying the development of Alzheimer’s disease, and reducing the impact and burden on patients and society.

## Figures and Tables

**Table 1:** The demographic and clinical baseline of the participants.

Variables	Training Set (N = 620)			Validation Set (N = 266)		
	With MCI	Without MCI	*p* Value	With MCI	Without MCI	*p* Value
Gender						
Female	45 (65.217)	378 (68.603)	0.666	26 (70.270)	164 (71.616)	1.000
Male	24 (34.783)	173 (31.397)		11 (29.730)	65 (28.384)	
Smoke	13 (18.841)	69 (12.523)	0.203	4 (10.811)	23 (10.044)	0.776
Alcohol	22 (31.884)	128 (23.230)	0.152	8 (21.622)	44 (19.214)	0.905
Age	58.072 ± 6.434	55.713 ± 6.236	0.005	61.000 ± 6.637	55.764 ± 6.296	<0.001
BMI	23.694 ± 2.811	24.298 ± 3.104	0.100	24.099 ± 2.396	24.464 ± 3.385	0.423
Glucose	5.392 ± 1.191	5.449 ± 1.155	0.705	5.811 ± 1.973	5.427 ± 1.313	0.260
TG	1.588 ± 0.758	1.575 ± 1.014	0.900	1.632 ± 0.781	1.781 ± 1.861	0.405
TCH	5.477 ± 0.836	5.372 ± 0.976	0.337	5.196 ± 0.932	5.397 ± 1.010	0.235
HDL	1.612 ± 0.398	1.792 ± 0.505	0.001	1.477 ± 0.379	1.777 ± 0.477	<0.001
LDL	3.145 ± 0.729	2.990 ± 0.718	0.098	3.046 ± 0.743	2.978 ± 0.732	0.605

* BMI, body mass index; TG, triglyceride; TCH, total cholesterol; HDL, high-density lipoprotein; LDL, low-density lipoprotein.

**Table 2:** MCI risk prediction model for the middle-aged and elderly.

Variables	β	Std Error	Z Value	*p* Value	OR	95%CI	
						Lower	Upper
Age	0.068	0.021	3.257	0.001	1.071	1.027	1.116
BMI	−0.090	0.047	−1.942	0.052	0.914	0.832	0.999
HDL	−0.920	0.318	−2.895	0.004	0.399	0.208	0.726
LDL	0.323	0.180	1.797	0.072	1.382	0.972	1.972
TDIA	0.177	0.090	1.970	0.049	1.193	0.992	1.428
DFGA	−1.141	0.559	−2.042	0.041	0.320	0.104	0.938
RDGA	0.122	0.044	2.749	0.006	1.130	1.036	1.233
DPGA	0.091	0.029	3.143	0.002	1.096	1.036	1.161

* TDIA, time difference of intestinal channel after meal; DFGA, dominant frequency of gastric channel after meal; RDGA, rhythm disturbance of gastric channel after meal; DPGA, dominant power ratio of gastric channel after meal.

## Data Availability

All datasets generated and analyzed for this study are included in the article/Supplementary Material.

## Supplementary Materials

The following supporting information can be downloaded at: https://www.mdpi.com/article/10.3390/biom12121861/s1, Table S1: Comparison of electrogastroenterography parameters of the participants.
