# Supplementary material for: Development of a Gastrointestinal-Myoelectrical-Activity-Based Nomogram Model for Predicting the Risk of Mild Cognitive Impairment
Source: Biomolecules. 2022 Dec 12;12(12):1861. doi: 10.3390/biom12121861 (PMC9775682; doi:10.3390/biom12121861)
Supplement: Supplementary file 1 [file biomolecules-12-01861-s001.zip › biomolecules-2047702-supplementary.pdf]

Supplementary Table S1. Comparison of electrogastroenterography parameters of the participants.

Variables	Training set (N = 620)			Validation set (N = 266)		
	With MCI	Without MCI	P Value	With MCI	Without MCI	P Value
MAGA	220.308±102.812	209.764±92.822	0.419	231.128±123.980	210.901±100.587	0.351
MFGA	3.328±0.231	3.430±0.304	0.001	3.385±0.427	3.421±0.311	0.629
RDGA	21.399±4.250	20.655±3.946	0.171	21.393±3.447	20.609±3.920	0.214
RAGA	78.569±38.147	74.943±32.527	0.452	84.514±46.165	74.811±34.842	0.228
TDGA	0.145±0.224	0.115±0.232	0.297	0.135±0.223	0.116±0.216	0.627
DFGA	2.904±0.229	2.986±0.273	**0.008**	2.863±0.257	2.983±0.299	**0.013**
DPGA	63.792±5.771	62.283±6.337	0.046	60.536±5.901	62.090±5.903	0.144
PGA	6.280±0.817	6.044±0.842	0.026	6.041±0.788	5.960±0.842	0.567
CPGA	92.927±6.748	93.217±6.614	0.737	92.180±6.347	92.627±7.333	0.700
MAIA	165.337±73.986	181.806±98.806	0.098	180.385±107.807	185.222±85.984	0.796
MFIA	11.568±2.001	12.045±2.063	0.066	11.020±1.753	12.256±2.221	<.001
RDIA	26.689±3.751	25.031±5.025	0.001	26.366±5.031	25.333±5.21	0.255
RAIA	61.018±27.057	67.831±35.817	0.061	66.074±38.1	68.998±31.202	0.66
TDIA	0.083±0.206	0.049±0.097	0.189	0.088±0.141	0.060±0.127	0.272
DFIA	10.496±2.150	10.705±2.363	0.455	9.619±1.770	10.766±2.528	0.001
DPIA	29.239±3.792	29.609±4.982	0.464	30.236±4.567	29.206±4.863	0.213
PIA	5.428±0.782	5.392±1.019	0.732	5.594±0.920	5.314±1.033	0.097
CPIA	91.43±8.385	90.869±10.351	0.611	94.314±4.987	91.289±10.581	0.006
MAGB	175.815±99.212	177.143±90.166	0.916	185.115±100.480	171.653±84.816	0.444
MFGB	3.404±0.279	3.476±0.321	0.05	3.464±0.289	3.474±0.321	0.836
RDGB	20.377±3.989	21.044±3.776	0.191	20.858±3.665	21.134±3.710	0.674
RAGB	62.623±36.546	63.748±31.916	0.808	65.372±36.390	61.657±30.557	0.559
TDGB	0.110±0.168	0.110±0.185	0.989	0.085±0.138	0.090±0.157	0.856
DFGB	2.979±0.264	2.994±0.250	0.658	2.990±0.261	2.998±0.259	0.858
DPGB	62.615±6.135	60.818±6.065	0.024	62.411±7.483	61.142±6.329	0.334
PGB	6.132±0.784	5.879±0.805	0.014	6.042±0.818	5.876±0.858	0.259
CPGB	91.485±6.695	90.535±7.287	0.274	89.226±8.890	89.748±8.198	0.739
MAIB	163.043±104.653	192.436±121.900	0.034	162.926±105.495	187.142±101.481	0.199
MFIB	12.601±2.574	12.874±2.248	0.404	11.873±1.999	12.936±2.479	0.005
RDIB	24.850±4.920	23.409±5.830	0.027	25.160±5.545	23.406±5.545	0.080
RAIB	61.101±38.312	72.284±44.237	0.027	60.068±36.979	70.499±37.575	0.119
TDIB	0.054±0.110	0.043±0.087	0.422	0.051±0.108	0.039±0.080	0.521
DFIB	11.545±2.926	11.605±2.723	0.873	10.839±2.396	11.736±2.850	0.045
DPIB	30.680±5.126	30.737±6.389	0.932	30.19±5.679	30.833±6.078	0.530
PIB	5.205±1.171	5.261±1.263	0.708	5.588±0.989	5.204±1.273	0.041
CPIB	81.947±17.750	82.909±17.902	0.673	86.556±12.349	83.165±17.860	0.154

* MAGA, mean amplitude of gastric channel after meal; MFGA, mean frequency of gastric channel after meal; RDGA, rhythm disturbance of gastric channel after meal; RAGA, reactive area of gastric channel after meal; TDGA, time difference of gastric channel after meal; DFGA, dominant frequency of gastric channel after meal; DPGA, dominant power ratio of gastric channel after meal; PGA, percentage of normal slow wave of gastric channel after meal; CPGA, coupling percent of gastric channel after meal; MAIA, mean amplitude of intestinal channel after

meal; MFIA, mean frequency of intestinal channel after meal; RDIA, rhythm disturbance of intestinal channel after meal; RAIA, reactive area of gastric intestinal after meal; TDIA, time difference of intestinal channel after meal; DFIA, dominant frequency of intestinal channel after meal; DPIA, dominant power ratio of intestinal channel after meal; PIA, percentage of normal slow wave of intestinal channel after meal; CPIA, coupling percent of intestinal channel after meal; MAGB, mean amplitude of gastric channel before meal; MFGB, mean frequency of gastric channel before meal; RDGB, rhythm disturbance of gastric channel before meal; RAGB, reactive area of gastric channel before meal; TDGB, time difference of gastric channel before meal; DFGB, dominant frequency of gastric channel before meal; DPGB, dominant power ratio of gastric channel before meal; PGB, percentage of normal slow wave of gastric channel before meal; CPGB, coupling percent of gastric channel before meal; MAIB, mean amplitude of intestinal channel before meal; MFIB, mean frequency of intestinal channel before meal; RDIB, rhythm disturbance of intestinal channel before meal; RAIB, reactive area of gastric intestinal before meal; TDIB, time difference of intestinal channel before meal; DFIB, dominant frequency of intestinal channel before meal; DPIB, dominant power ratio of intestinal channel before meal; PIB, percentage of normal slow wave of intestinal channel before meal; CPIB, coupling percent of intestinal channel before meal.
